# Face Recognition with Multi-Resolution Spectral Feature Images

**DOI:** 10.1371/journal.pone.0055700

**Published:** 2013-02-13

**Authors:** Zhan-Li Sun, Kin-Man Lam, Zhao-Yang Dong, Han Wang, Qing-Wei Gao, Chun-Hou Zheng

**Affiliations:** 1 School of Electrical Engineering and Automation, Anhui University, Hefei, China; 2 Department of Electronic and Information Engineering, Hong Kong Polytechnic University, Hong Kong, China; 3 Center for Intelligent Electricity Networks, University of Newcastle, Newcastle, Australia; 4 School of Electrical and Electronic Engineering, Nanyang Technological University, Singapore, Singapore; Universitat Rovira i Virgili, Spain

## Abstract

The one-sample-per-person problem has become an active research topic for face recognition in recent years because of its challenges and significance for real-world applications. However, achieving relatively higher recognition accuracy is still a difficult problem due to, usually, too few training samples being available and variations of illumination and expression. To alleviate the negative effects caused by these unfavorable factors, in this paper we propose a more accurate spectral feature image-based 2DLDA (two-dimensional linear discriminant analysis) ensemble algorithm for face recognition, with one sample image per person. In our algorithm, multi-resolution spectral feature images are constructed to represent the face images; this can greatly enlarge the training set. The proposed method is inspired by our finding that, among these spectral feature images, features extracted from some orientations and scales using 2DLDA are not sensitive to variations of illumination and expression. In order to maintain the positive characteristics of these filters and to make correct category assignments, the strategy of classifier committee learning (CCL) is designed to combine the results obtained from different spectral feature images. Using the above strategies, the negative effects caused by those unfavorable factors can be alleviated efficiently in face recognition. Experimental results on the standard databases demonstrate the feasibility and efficiency of the proposed method.

## Introduction

Over the past decades, face recognition technology has become one of the most important biometric fields [1], [2]. Due to its relative high recognition accuracy and low intrusiveness, it has been widely applied in various scenarios, such as information security, law enforcement, surveillance, and so on. Many algorithms have been developed to address various problems with face recognition [3]–[5], such as expression variation, pose variation, 3D face recognition, multi-modal 2D+3D face recognition, multi-biometric feature fusion, etc.

In recent years, face recognition for the one-sample-per-person problem has attracted many researchers to this research branch. There are two main reasons for this. On the one hand, this problem is very common in some existing application scenarios, such as law enforcement, driver’s license, passport and identity card identification, where only a single frontal-view image per person is available. Therefore, it is necessary to develop some more efficient and effective algorithms to make face recognition techniques applicable to these situations. On the other hand, storing only one sample per person in a database can very effectively reduce the costs of sample collection, storage and computation [6].

Different approaches have been proposed for the one-sample-per-person face recognition problem [6], [7]. Principal component analysis (PCA) is a widely used statistical signal processing technique [8], [9]. Various extensions of PCA have been proposed to solve the one-sample-per-person problem [10]–[12]. Instead of using global features, a representation extracted from patches is proposed in [13] for face recognition with a single exemplar image per person. A prominent advantage of using local representations is its fair robustness to variations in lighting, expression and occlusion. Multiple-feature fusion is also an effective approach for the one-sample-per-person face recognition problem. A combination of the frequency invariant features and the moment invariant features [14], and a fusion of the directionality of edges and the intensity facial features [15], are proposed for face recognition with a single training sample. Instead of using 2D representation, a 3D model-based method is an important approach to the one-sample-per-person face recognition problem. In [7], a good review of state-of-the-art 3D facial reconstruction methods [16], [17] for face recognition based on a single 2D training image per person is provided. Generally speaking, a common approach to deal with the one-sample-per-person face recognition problem is to enlarge the training set by constructing new representations [18]–[20] or by generating novel views [21].

Linear discriminant analysis (LDA) is a well-known technique for feature extraction and dimensionality reduction that has been used widely in numerous applications. To overcome the so-called singularity problem, a new type of LDA, called two-dimensional LDA (2DLDA), has been proposed and applied to image recognition in recent years [22]–[24]. Compared to the classical LDA, an obvious difference with 2DLDA is that the data is represented in a matrix form instead of a vector form. 2DLDA and its variants have attracted much attention in the past several years because of its advantages in dealing with the singularity problem and in computational cost. Although 2DLDA represents data in a matrix form, it cannot be directly applied to solving the one-sample-per-person problem because the within-class scatter matrix is a zero matrix, which makes it unstable. In [25], the difference between the original image and the reconstructed image obtained in using singular value decomposition (SVD) was found to be able to reflect the variations in the within-class images, to an extent. Therefore, the original image and the reconstructed image, instead of the training images only, are used together to compute the within-class scatter matrix and the between-class matrix. The discriminant feature obtained by 2DLDA has been demonstrated to be superior to some existing methods [10], [26], [27].

Information in the frequency domain is useful in image classification. In [28], a global feature of a scene, named “spatial envelope”, is proposed by exploring the dominant spatial structure of a scene. For this global feature, the global energy spectrum is used to develop spectral signatures for each scene category. To capture the textural characteristics of the image in the frequency domain, a variant of the global energy feature is presented further in [29], which explores the statistics of the co-occurrence matrix.

Although the spectral feature is specially designed for scene classification, in this paper we present a spectral representation of face images and apply this representation to the one-sample-per-person problem. One issue with the one-sample-per-person problem is that the number of training sample available is too few. In this paper, multi-resolution spectral images are extracted and used as representations of training face images by means of a method similar to [28], thereby enlarging the size of the training set greatly. We find that, among these spectral feature images, features extracted from some specific orientations and scales using 2DLDA are not sensitive to variations of illumination and expression. Inspired by this finding, in our algorithm the spectral features are used as a robust representation of faces. As we do not know exactly which orientations and scales are robust for all testing images, an alternative approach is to use all of these filters in the decision-making process. In our method, each of the filters will form one weak classifier. The strategy of classifier committee learning (CCL) is designed further to combine the results obtained from different spectral feature images to determine the classes of the testing images. With the strategy of CCL, on the one hand, most of the correct categorizations can be retained. On the other hand, it is not necessary for us to choose the optimal filters, which is a very difficult task for the one-sample-per-person problem. Using the above strategies, the negative effects caused by those unfavorable factors, such as variations of illumination and facial expression, can be alleviated greatly in face recognition. Experimental results on some standard databases demonstrate the feasibility and efficiency of the proposed method.

## Methodology

**Figure 1:**
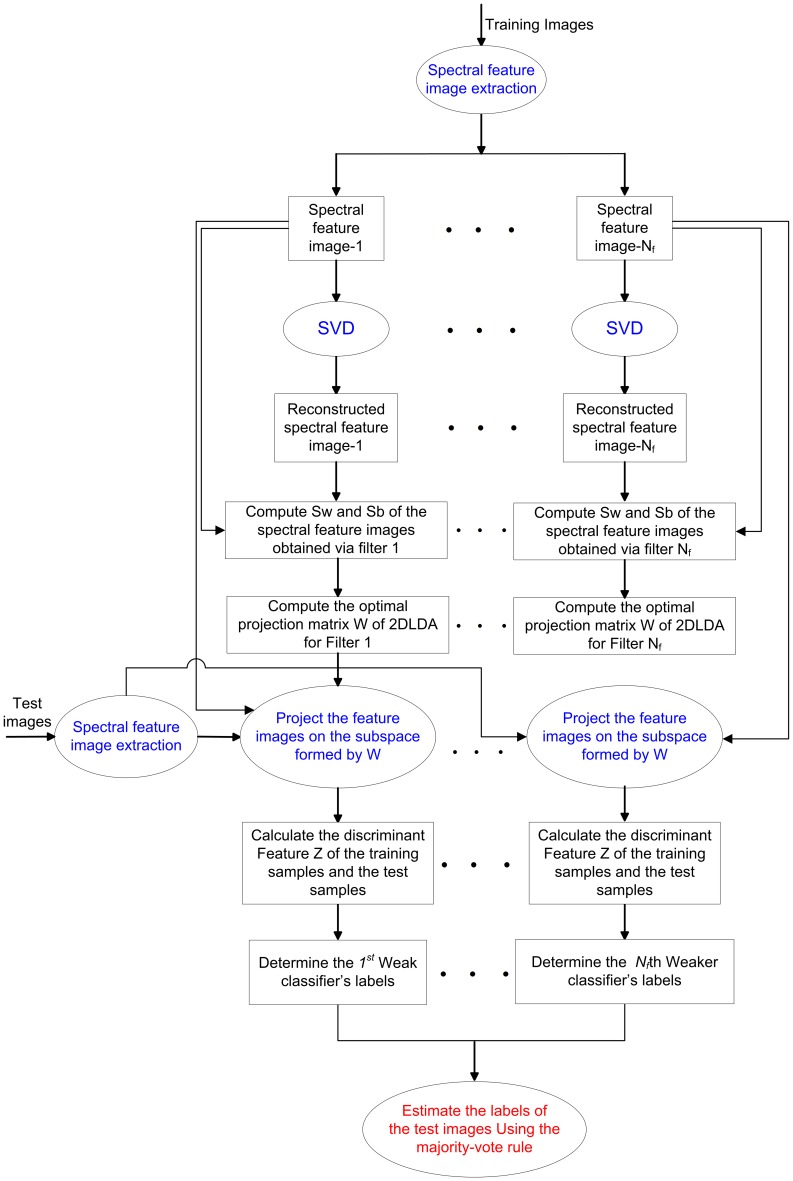
Flowchart of the multi-resolution spectral feature image based 2DLDA ensemble algorithm.

Fig. 1 shows the flowchart of our multi-resolution spectral feature image-based 2DLDA ensemble algorithm. There are three main parts to the proposed method: spectral feature image extraction, discriminant feature extraction, and the combination of weak classifiers. A detailed description of each of these three parts is presented in the following subsections.

### Spectral Feature Image Representation

**Figure 2:**
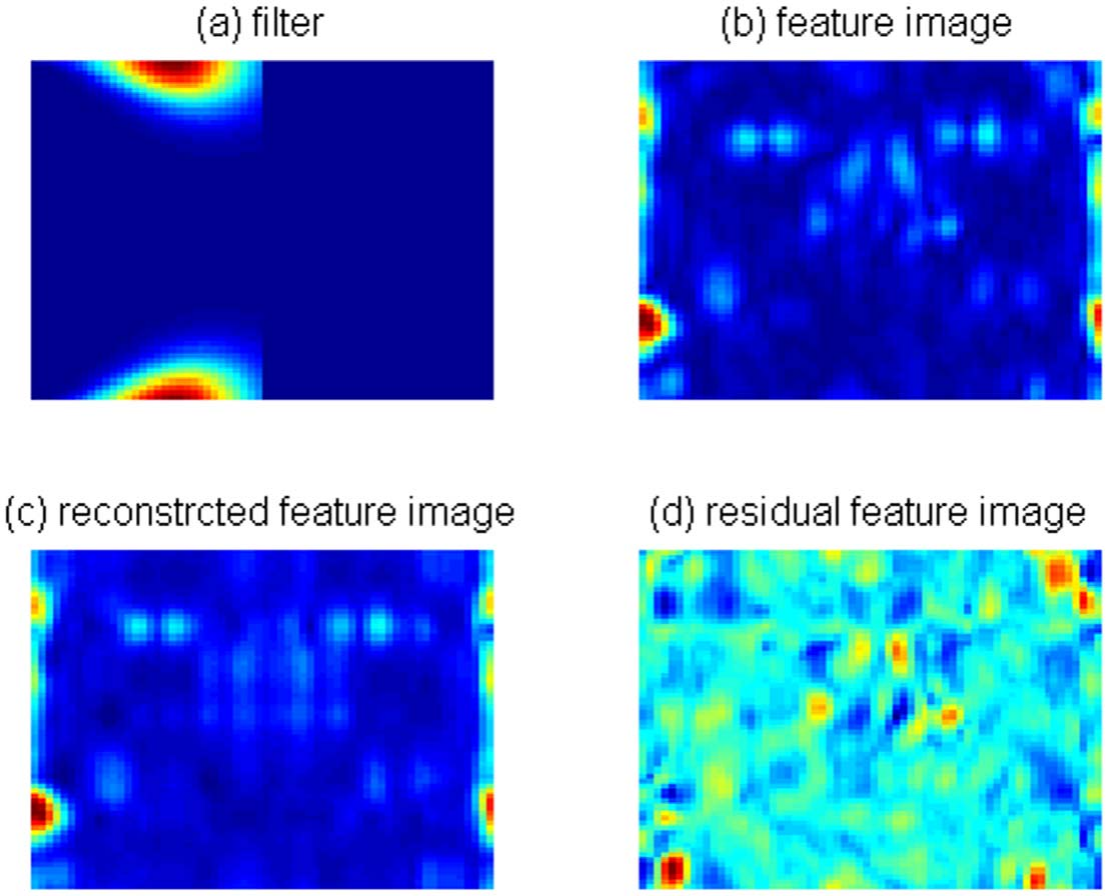
An example of spectral feature image extraction: (a) the filter, (b) the spectral feature image, (c) the reconstructed feature image, and (d) the residual feature image.

Assume that there are 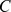 training images 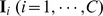 with size 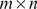, and that each belongs to one subject. We first extract the spectral feature images of each training image. The image is first pre-filtered to reduce the effect of illumination, using a local normalization method of intensity variance as follows [28]:
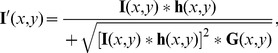
(1)where 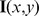 and 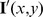 are pixel intensities before and after pre-filtering, respectively, 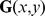 is an isotropic low-pass Gaussian spatial filter with a radial cut-off frequency at 0.015 cycles/pixel, and 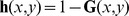.  is a constant that helps suppress noise in low-frequency regions. Next, a set of Gabor filters with 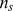 scales and 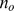 orientations is applied on the Fourier transform of the prefiltered image [28]:



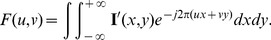
(2)Finally, the amplitude of the resulting image is computed as the spectral feature image. As a result, for the given 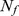 (i.e. 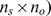 filters, 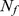 spectral feature images can be obtained for each training sample. Given the filter shown in Fig. 2(a), the computed spectral feature image, the reconstructed feature image and the residual feature image are shown in Figs. 2(b) - Fig. 2(d), respectively. The spectral feature images are then used as the inputs of 2DLDA to obtain the most discriminant projection vectors.

### Discriminant Feature Extraction

Having generated the spectral feature images based on the 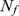 Gabor filters for all the training face images, we can obtain 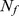 optimal projection subspaces via 2DLDA [25]. Subsequently, 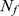 sets of discriminant feature can be derived by projecting the feature image onto the optimal projection subspace. Denote 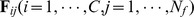 as the spectral feature image of the training image 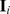 obtained by using the 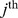 filter. The unitary matrices 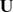, 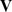 and the diagonal matrix 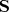 constitute the SVD of 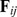, i.e.,
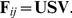
(3)


If the first 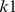 SVD basis images are used, the corresponding reconstructed feature image can be given as follows:
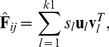
(4)where the singular values 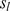 are the diagonal elements of 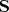, and 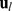 and 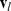 are the 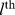 column of 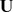 and 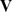, respectively. Given the spectral feature images 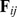 and the reconstructed feature images 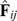, the mean feature image 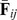 and the global mean 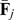 of the 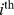 2DLDA are defined as follows:
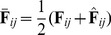
(5)and



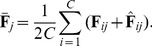
(6)Then, the between-class matrix 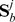 and the within-class scatter matrix 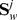 can be computed as follows:
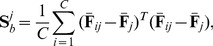
(7)

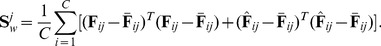
(8)


Denote 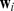 as the eigenvectors of the following generalized eigenvalue problem:
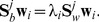
(9)where 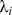 are the eigenvalues. The optimal project matrix 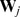 is composed of the eigenvectors associated with the first 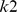 largest eigenvalues, i.e.,
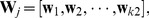
(10)which maximize the following criterion:



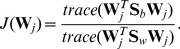
(11)The discriminant features 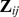 can be computed by projecting the spectral feature image 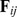 onto the subspace spanned by 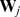, i.e.,
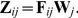
(12)


As a result, we can obtain 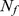 discriminant features 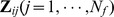 for each training image 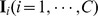.

### Combining the Weaker Classifiers

From Fig. 2, we cannot directly observe whether an extracted spectral feature image is sensitive to the variations of illumination or expression. As an alternative, we investigate the sensitivity by checking the predicted labels of test samples for different filters. Given a test image 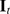, we first extract its spectral feature images 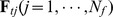, and then compute the discriminant features 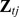 using (12). With the discriminant features of the training images 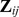 and that of the test image 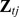, the nearest-neighbor classifier is used here to assign a class label to 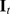. The test image 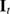 belongs to the 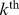 class if
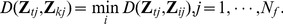
(13)


For a subject with different expression, illumination and occlusion, Table 1 shows the predicted labels of test samples for different filters when the first image is used as the training sample. We see that the labels can be predicted correctly for the features extracted from spectral feature images at particular scales and orientations. Unfortunately, these orientations and scales are not consistent for the different test samples. That is to say, we cannot predict which scales and orientations are not sensitive to variations of illumination and expression for different face images.

**Table 1:** The predicted labels of the test images for different filters when the first image is used as the training sample.

test imagefilter	(b)	(c)	(d)	(e)	(f)	(g)	(h)	(i)	(j)	(k)
1	10	10	4	1	15	14	8	14	6	1
2	14	15	14	10	9	6	9	15	10	14
3	13	10	1	1	15	15	2	15	6	5
4	6	10	1	1	2	14	2	2	11	1
5	1	7	1	1	1	14	7	12	14	7
6	1	1	1	1	1	1	4	1	2	1
7	7	1	1	1	2	1	2	1	2	1
8	5	13	1	1	1	1	2	6	12	5
9	6	13	12	1	6	1	2	6	6	2
10	6	12	1	6	1	5	6	1	10	6
11	4	10	5	4	9	5	9	15	13	14
12	12	3	4	11	11	11	11	2	6	5
13	5	14	1	5	14	14	4	4	6	3
14	13	13	14	6	3	1	13	13	10	3
15	6	15	1	15	11	5	7	1	7	6
16	5	14	1	1	1	7	4	13	3	14
17	1	3	3	1	1	1	1	1	7	14
18	1	3	3	12	9	9	1	6	12	3
19	12	12	5	14	3	14	3	3	9	12
20	1	3	1	3	3	1	3	1	12	3
21	8	3	3	3	8	1	8	15	8	10
L(MR_2DLDA)	1	3	1	1	1	1	2	1	6	1
L(2DLDA)	1	1	1	2	6	15	2	6	2	2
L(True)	1	1	1	1	1	1	1	1	1	1

Since we cannot select the optimal scales and orientations, an alternative approach is to use all of these filters in the decision process. We construct one weaker classifier for each filter, as shown in (13). As a result, for each test sample, 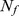 weaker classifiers are formed by means of the spectral features extracted via 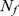 filters. Finally, a classifier-combination strategy is adopted finally to determine the class label of the test image. Max rule, min rule, median rule, and majority-vote rule are commonly used classifier-combination strategies [30]. As the outputs of the weaker classifiers are the class labels of the test images, the majority-vote rule is the most suitable strategy to combine these outputs. To count the votes received from the weaker classifiers, a binary-valued vector is defined. If 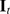 belongs to the 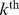 class, the class label vector of 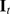 obtained via the 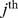 weaker classifier can be given as follows:
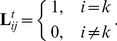
(14)


It can be seen from (14) that one binary-valued vector can be obtained for each weaker classifier. Further, we sum these vectors to obtain the number of votes for each class as follows:
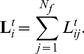
(15)


Each element of 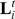 denotes the number of votes of each class. The test sample 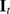 belongs to the class with the maximum number of votes. For example, the label of 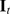 is 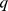 if the 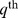 element of 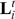 is maximum.

The labels (L(MR_2DLDA)) determined via the majority-vote rule are tabulated in Table 1. As a comparison, the labels (L(2DLDA)) predicted through 2DLDA are also shown in Table 1. It can be seen that, comparing to 2DLDA, more labels are predicted correctly using the proposed method. There are two main reasons for this. One the one hand, the spectral features extracted on some scales and orientations are not sensitive to variations of illumination and expression. As shown in Table 1, 2DLDA cannot predict the labels correctly for some test samples, whiles these labels are correctly assigned by some weaker classifiers. This provides the possibility to predict the label of the test samples correctly. On the other hand, although we have no way of choosing the optimal filters, as discussed previously, the majority-vote rule can find the correct class attributes of the test samples when the spectral features extracted on a large percentage of scales and orientations are not sensitive to variations of illumination and expression. Certainly, we can also see that not all the labels of the test images are predicted correctly using our proposed method. Therefore, the strategies adopted in the proposed method can only alleviate the negative effects caused by variations of illumination and expression to some extent.

## Experimental Results

### Databases and Experiment Set-Up

We evaluate the performance of our proposed method on seven standard databases: Yale face database [31], ORL face database [32], Extended Yale Face database B, PIE database, FERET face database, AR database, and LFWA database [33], [34].

The Yale face database contains 165 grayscale images of 15 individuals. Each individual has 11 images that are different in expressions (happy, normal, sad, sleepy, surprised, and winking), in lighting conditions (left-light, center-light, right-light), and in facial details (with/without glasses) [31].

There are ten different images for each of the 40 distinct subjects in the ORL face database. For some subjects, the images were taken at different times and with different lighting conditions, facial expressions (open/closed eyes, smiling/not smiling) and facial details (with/without glasses) [35].

The Extended Yale Face database B has 38 individuals and around 64 near frontal images under different illuminations per individual. The PIE database contains images of 68 individuals. There are about 170 images for each individual under 5 near frontal poses (C05, C07, C09, C27, C29), 43 different illumination conditions, and with 4 different expressions [36]. In the FERET face database, there are 3,280 gray-level frontal-view face images of 1010 persons. For this database, those subjects with more than 10 images are selected for testing in the experiments. For the AR database, the face images of 100 subjects are used in the experiments. Each subject contains 14 face images that are different in illumination and expression.

LFWA is a database of face photographs designed for studying the problem of unconstrained face recognition. All the face images have been aligned via commercial face-alignment software by the provider. Further, like other datasets, the face images are manually cropped to remove the backgrounds. As the whole database is fairly large, and the face images are manually cropped, we have selected only a subset from the database in the experiments. Those individuals with 30 or more images were selected for the experiments. In the selected dataset, there are 34 individuals in total.

Except for the LFWA database, all the face images are manually aligned and cropped by other researchers. To investigate the influence of the image size on the recognition performance, we use both the Yale face database and the ORL face database, which have two sets of data with different image sizes. From the Yale face database, the images of size 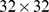 (denoted as Yale_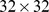) and 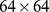 (denoted as Yale_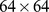) were used in the experiments. For the ORL face database, the images of size 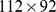 (denoted as ORL_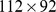) and 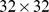 (denoted as ORL_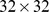) were utilized in the evaluation. The Extended Yale Face database B, the PIE database, the FERET face database, and the AR database have far larger numbers of face images than the other two databases. Therefore, they can be used to investigate the performance of the algorithms on large databases. The image sizes of the Extended Yale Face database B, the PIE database, the FERET face database, and the AR database are 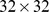, 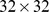, 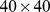, and 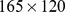, respectively (denoted as YaleB_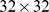, PIE_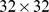, FERET_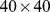, and AR_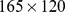, respectively). The above datasets are publicly available from [32], [34], [37].

### 0.1 Experiments

To verify the performance of our proposed method (denoted as MR_2DLDA), we compare it to four other face recognition methods designed for the one-sample-per-person problem. The four methods are the E(PC)^2^ A method [10], the block-based Fisher LDA method (denoted as BFLDA) [26], the generalized eigenface method (denoted as GE) [27], the 2DLDA [25].

As in [38], three scales are employed for the filter transfer functions. The respective numbers of orientations for the three scales (NOS) are set at 8, 8, and 4 (denoted as [8 8 4]). Our experiments have shown that a satisfactory performance can generally be achieved when the parameters of the filter transfer functions are set around the values suggested in [38]. Table 2 shows the recognition rates of MD_2DLDA on the datasets Yale_32×32 and ORL_32×32, with the first image of each subject in the databases used as training samples and different numbers of Gabor filters are used. As only one training sample is used for each distinct subject, traditional parameter-selection methods, such as cross validation, cannot be used to choose the optimal parameters. It can be seen from Table 2 that the classification results are close for different parameters, i.e., the parameter variation around [8 8 4] only has slight influence on the classification performance. Therefore, for simplicity, in all the following experiments, the respective numbers of orientations for the three scales are set at 9, 8, and 4 (denoted as [9 8 4]), i.e., twenty-one filters are used in our proposed method. Similar results can be obtained when other parameters are adopted in the experiments.

**Table 2:** The recognition rates (%) of MR_2DLDA on the datasets Yale_32×32 and ORL_32×32, with the first image of each subject in the databases used as the training sample, when different numbers of Gabor filters are used.

NOS	[6 8 4]	[7 8 4]	[8 8 4]	[9 8 4]	[10 8 4]
Yale_32×32	67.33	69.33	69.33	74.66	71.33
ORL_32×32	72.50	73.06	71.39	71.39	73.33
NOS	[8 6 4]	[8 7 4]	[8 8 4]	[8 9 4]	[8 10 4]
Yale_32×32	70.00	72.67	69.33	70.00	72.00
ORL_32×32	74.17	72.78	71.39	72.78	73.61
NOS	[8 8 2]	[8 8 3]	[8 8 4]	[8 8 5]	[8 8 6]
Yale_32×32	66.00	67.33	69.33	70.00	68.67
ORL_32×32	72.50	72.22	71.39	73.61	72.78

**Figure 3:**
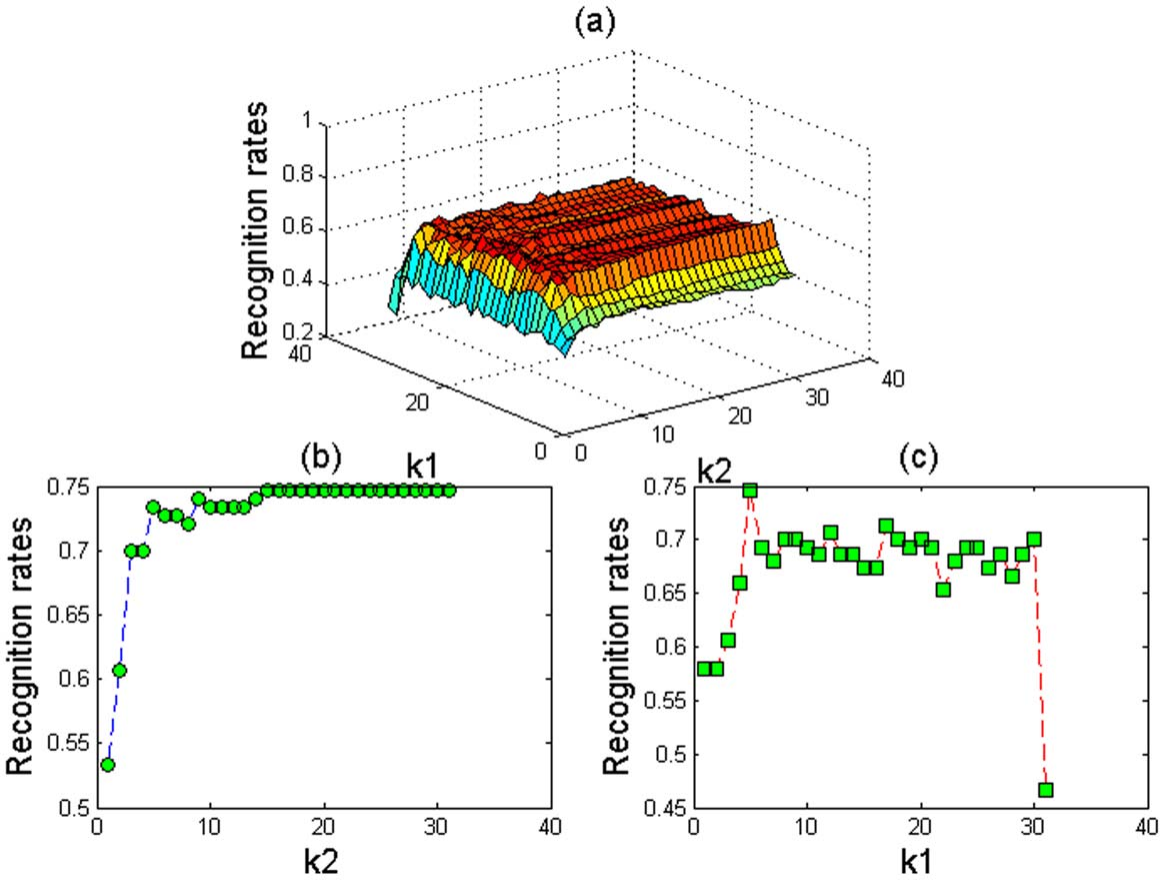
The recognition rates of our proposed method with different values of 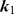 and 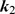 based on the Yale_32×32 database: (a) 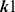 and 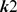 vary in the interval **[1]**–**[31]**, (b) 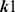 is set at 5 and 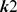 varies in the interval **[1]**–**[31]**, and (c) 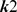 is set at 20 and 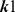 varies in the interval **[1]**–**[31]**.

**Figure 4:**
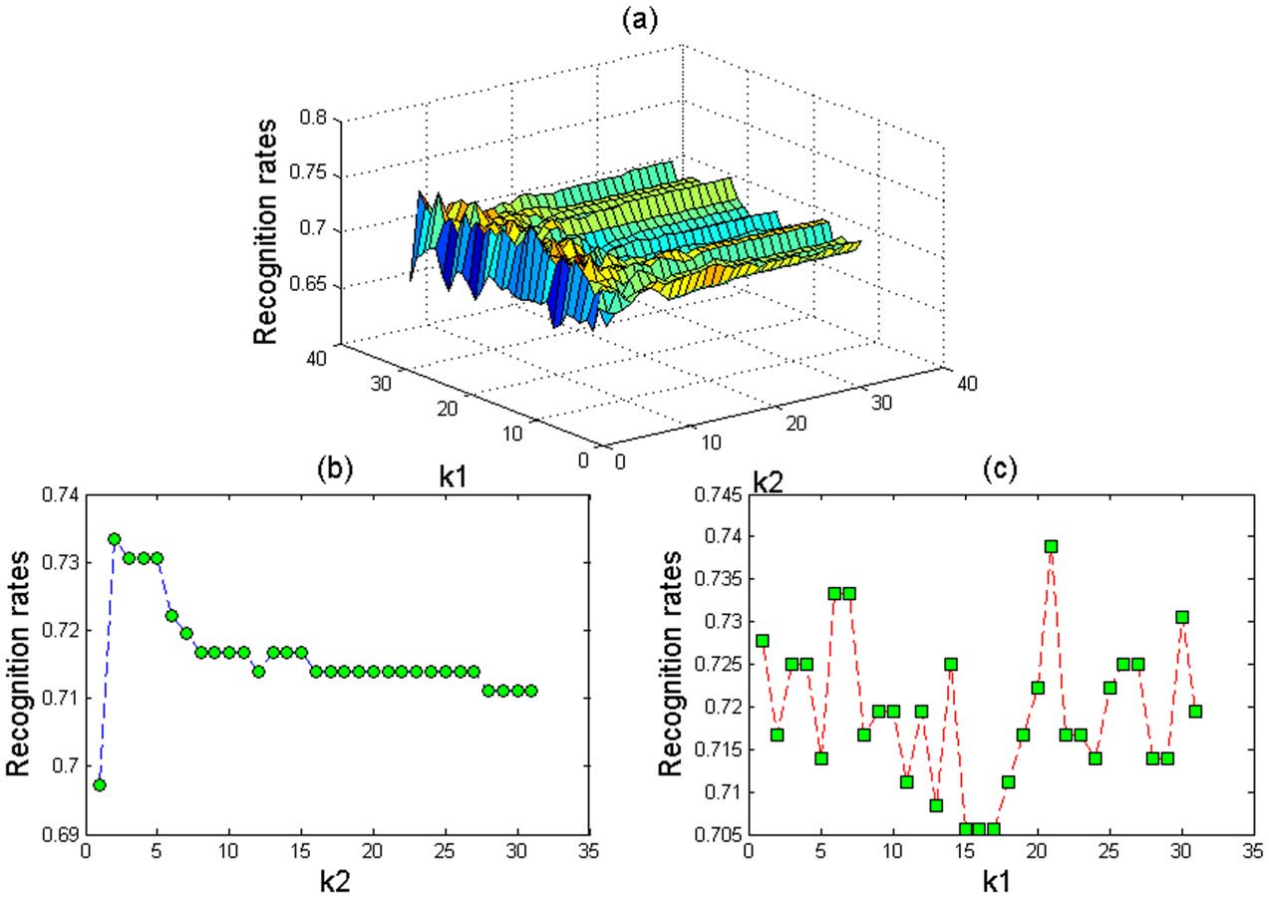
The recognition rates of our proposed method with different values of 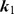 and 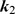 based on the ORL_32×32 database: (a) 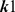 and 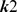 vary in the interval [**1]**–**[31]**, (b) 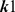 is set at 5 and 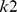 varies in the interval [**1]**–**[31]**, and (c) 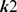 is set at 20 and 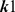 varies in the interval [**1]**–**[31]**.

It is also difficult to find the optimal values for the parameters 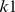 and 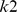 in the one-sample problem. Taking the dataset Yale_32×32, for example, Fig. 3(a) shows the recognition rates when 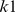 and 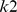 vary in the interval [1]–[31]. Fig. 3(b) shows the results when 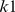 is 5 and 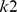 varies in the interval [1]–[31]. It can be seen that the performance becomes stable when  is larger than 15. Fig. 3(c) shows the results when 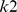 is 20 and 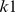 varies in the interval [1]–[31]. We can see that the recognition rate increases gradually when 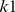 varies from 1 to 5, and then the recognition rate decreases with some fluctuations when 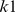 varies from 6 to 31. Fig. 4 shows the corresponding results based on the dataset ORL_32×32. We can conclude that a better result is obtained when 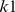 and 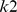 are set at 5 and 20, respectively. Since we are unable to identify the optimal values of the parameters 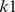 and 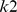 via the parameter-selection methods, in all the following experiments, 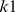 and 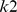 are set at 5 and 20, respectively.

The performance of our proposed method is compared to four different face recognition methods. We follow the same experimental set-ups as used in [25]: the first image of each subject is used as the training sample, while the remaining images are used as the test samples. We first perform a set of experiments on the datasets Yale_32×32 and ORL_112×92 to compare the recognition performances of the five different face recognition methods. Table 3 shows the top 1 recognition rates (%) of the five methods based on the two datasets. Note that the experimental results of E(PC)^2^ A, BFLDA, GE and 2DLDA are given by [25]. For the dataset Yale_32×32, Table 3 shows that our proposed method can achieve much higher recognition accuracy than the other four methods. For the dataset ORL_112×92, compared to the other four methods, the recognition rate of our proposed method is between 8% and 40% higher than the other four methods.

**Table 3:** The recognition rates (%) of five different face recognition methods on the datasets Yale_32×32 and ORL_112×92, with the first image of each subject used as the training sample.

	Yale_32×32	ORL_112×92
E(PC)^2^ A	18.67	44.17
BFLDA	32.00	70.83
GE	23.33	46.39
2DLDA	34.67	75.56
MR_2DLDA	74.67	83.89

As 2DLDA has been demonstrated to have a superior performance as compared to the other three methods, we therefore compare the performances of 2DLDA and MR_2DLDA only, based on the datasets Yale_64×64, ORL_32×32, YaleB_32×32, PIE_32×32, FERET_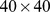, AR_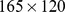, and LFWA_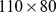 (see Table 4). For 2DLDA, the parameter 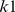 is set at 3, as is in [25]. The parameter 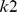 is set at 6 in terms of the experimental results shown in Fig. 8 of [25]. It can be seen from Table 4 that our proposed method can achieve much higher recognition accuracy than 2DLDA on Yale_64×64 and YaleB_32×32. Also, our proposed method has recognition rates about 10%, 17%, 20%, 31% and 13% higher than 2DLDA on ORL_32×32, PIE_32×32, FERET_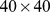, AR_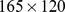, and LFWA_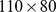, respectively. Furthermore, we can see from Tables 3 and 4 that, with our proposed method, the larger the image size, the higher the recognition rates will generally be, and vice versa.

**Table 4:** The recognition rates (%) of 2DLDA and MR_2DLDA on the datasets Yale_64×64, ORL_32×32, YaleB_32×32, PIE_32×32, FERET_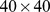, and AR_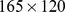 with the first image of each class used as the training sample.

	2DLDA	MR_2DLDA
Yale_64×64	25.33	80.00
ORL_32×32	61.94	71.39
YaleB_32×32	29.92	74.75
PIE_32×32	5.75	22.61
FERET_40×40	25.13	45.24
AR_165×120	32.77	63.85
LFWA_110×80	12.03	25.13

We noticed that some classification results on the ORL database and the Yale database are also reported for a multiple-feature method (denoted as MFM) [14]. Here, we can present only a rough comparison because the image sizes and the experimental set-ups are different for our MR_2DLDA method and the MFM method. It can be seen from Tables 9 and 10 in [14] that, the classification rates of the ORL database and the Yale database are 71% and 0.69%, respectively, when the first image of each individual is used as the training sample. However, we can see from Table 4 that the corresponding classification rates of the MR_2DLDA method are 71.39% and 80%, respectively. Moreover, in [14], we noted that the image sizes of the ORL database and the Yale database are 92×92 and 128×128, respectively, which are larger than the image sizes (32×32 and 64×64) used in evaluating the MR_2DLDA method. In terms of the conclusion we drew from Tables 3 and 4, a higher recognition accuracy generally can be achieved for the MR_2DLDA method if the databases with larger image sizes are available. In general, the MR_2DLDA method has a classification performance that is competitive with the MFM method on both the ORL database and the Yale database.

**Figure 5:**
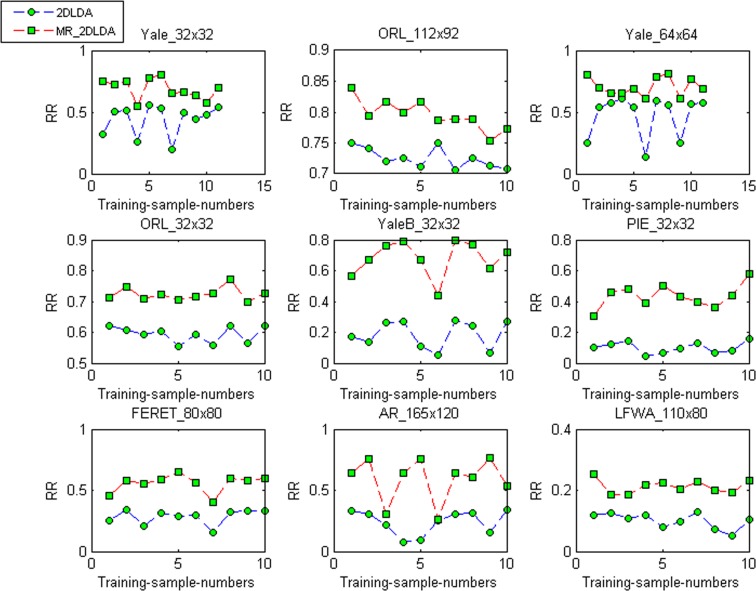
The recognition rates (RR) of 2DLDA and MR_2DLDA when different face images are used as the training samples.

Furthermore, to investigate the influence of different training samples on the recognition performance, each face image of every class is used as the training sample for the datasets ORL_32×32, ORL_112×92, Yale_64×64 and ORL_32×32. For the datasets, YaleB_32×32, PIE_32×32, FERET_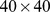, AR_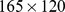, and LFWA_110×80, one face image is randomly selected from every class and used as the training sample. The trials are performed for ten times. Fig. 5 shows the recognition rates of 2DLDA and MR_2DLDA, respectively, on 9 datasets when different face images are used as the training samples. It can be seen that MR_2DLDA has a better recognition performance than 2DLDA on these datasets. Tables 5 and 6 show the mean (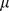), standard deviation (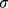), and the ratio (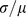) of the top 1 recognition accuracies (%) for 2DLDA and MR_2DLDA when different face images are used as the training samples, respectively. It can be seen that the mean recognition rates of MR_2DLDA are higher than those of 2DLDA by about 7
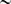
49%. These two methods have similar 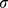 and 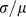 values on the datasets ORL_112×92 and ORL_32×32. However, MR_2DLDA has lower 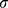 and 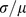 values on the datasets Yale_32×32, Yale_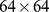, and LFWA_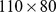 than those of 2DLDA. Moreover, MR_2DLDA has lower 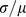 values on the datasets YaleB_32×32, PIE_32×32, FERET_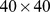, and AR_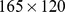 than those of 2DLDA. Therefore, we can conclude that MR_2DLDA is more robust than 2DLDA to the training samples used. We also can see that the performance of MR_2DLDA is obviously better than that of 2DLDA on four large datasets YaleB_32×32, PIE_32×32, FERET_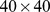, and AR_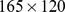. In addition, the experimental results of the two methods in Tables 5 and 6 again verify that, the larger the image size, the higher the recognition rates will generally be, and vice versa.

**Table 5:** The mean (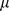), standard deviation (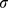), and the ratio (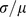) of the the recognition rates (%) for 2DLDA when different face images are used as the training samples.

	*μ*	*σ*	σ/*μ*
Yale_32×32	43.94	12.37	28.15
ORL_112×92	72.50	1.68	2.31
Yale_64×64	47.27	17.03	36.02
ORL_32×32	59.39	2.61	4.39
YaleB_32×32	18.64	8.83	47.38
PIE_32×32	10.75	3.73	36.78
FERET_40×40	28.26	6.19	21.91
AR_165×120	23.90	9.88	41.36
LFWA_110×80	10.11	2.58	25.49

**Table 6:** The mean (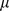), standard deviation (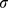), and the ratio (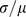) of the recognition rates (%) for MR_2DLDA when different face images are used as the training samples.

	*μ*	*σ*	σ/*μ*
Yale_32×32	68.97	8.30	12.03
ORL_112×92	79.56	2.44	3.06
Yale_64×64	70.48	7.53	10.68
ORL_32×32	72.36	2.20	3.03
YaleB_32×32	67.93	11.45	16.86
PIE_32×32	43.40	7.67	17.67
FERET_40×40	55.66	7.46	13.40
AR_165×120	59.07	17.88	30.27
LFWA_110×80	21.23	2.21	10.39

## Discussion

**Figure 6:**
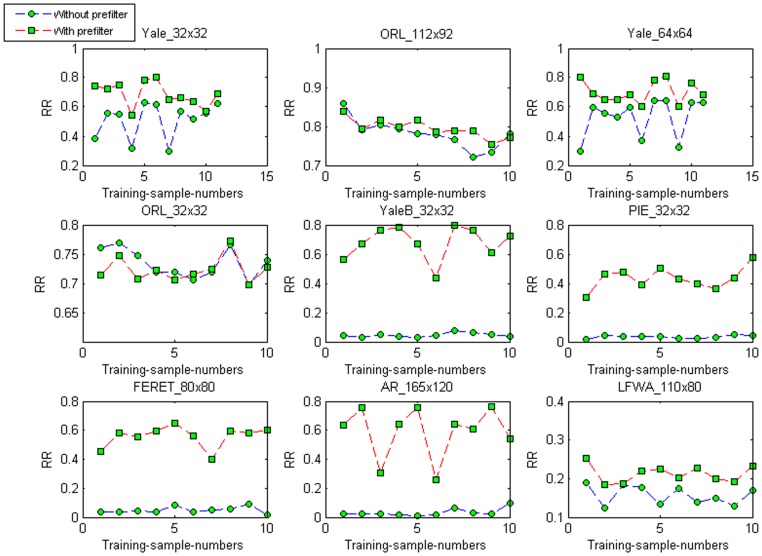
The recognition rates (RR) of MR_2DLDA with and without pre-filtering when each image of every class is used as the training sample.

Pre-filtering is an important step in the MR_2DLDA method. Fig. 6 shows the recognition rates of MR_2DLDA with and without pre-filtering, respectively, when different face images are used as the training samples. Table 7 shows the mean (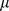), the standard deviation (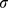), and the ratio (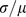) of the recognition rates (%) for MR_2DLDA when pre-filtering is not employed. We can see from Tables 6 and 7 and from Fig. 6 that the performances decrease greatly on the datasets Yale_32×32, Yale_64×64, YaleB_32×32, PIE_32×32, FERET_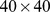, AR_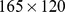, and LFWA_110×80, especially for the five large datasets. Although there is little change on the two ORL datasets, we can conclude, in general, that pre-filtering is an important step in the MR_2DLDA method.

**Table 7:** The mean (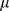), standard deviation (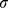), and the ratio (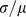) of the recognition rates (%) for MR_2DLDA without pre-filtering when different face images are used as the training samples.

	*μ*	*σ*	σ/*μ*
Yale_32×32	51.39	12.13	23.60
ORL_112×92	78.08	3.76	4.81
Yale_64×64	53.21	13.34	25.07
ORL_32×32	73.44	2.59	3.53
YaleB_32×32	4.47	1.46	32.76
PIE_32×32	3.38	1.05	31.07
FERET_40×40	4.88	2.15	44.04
AR_165×120	3.33	2.58	77.47
LFWA_110×80	15.67	2.48	15.81

Traditional parameter-selection methods, such as cross validation, cannot be used to choose the optimal parameters for face recognition in the case of the one-sample-per-person problem. For our proposed method, the parameters 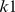, 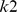 and 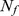 can only be determined experimentally. This problem is also encountered by other existing face recognition algorithms in the one-sample-per-person case. How to find the optimal parameter values is still to be investigated in our future work.

A heavy computation burden is a common problem in the CCL algorithms. The proposed method also has a higher computation cost than does 2DLDA. The reason for this is that the feature extraction and classification are performed based on each individual filter, i.e., 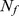 times in all. The computation time can be reduced by selecting only some of the filters, instead of using all the filters in the experiments. However, it remains a difficult problem to find an efficient criterion to select those filters that are efficient for all datasets.

An argument of CCL is that the number of samples is increased when the number of weaker classifiers is increased via the rand subspace method, or another such method. For our proposed method, we can increase the training set size by constructing the spectral images of the training samples. Then, the features are extracted by using the conventional LDA algorithms, and the test samples are classified using the nearest-neighbor algorithm. The results are poor for the cases cited in this paper. Another possible variant of the proposed method is that, instead of using 2DLDA, other LDA algorithms such as the well-known regularized discriminant analysis [39], etc., can also be embedded in the proposed approach, thereby substituting 2DLDA. As 2DLDA has been demonstrated to have a superior performance as compared to the other methods, there is no need to present more experimental results here.

Although our proposed method is specifically designed for face recognition in the one-sample-per-person problem, it can also be extended to deal with cases with more than one sample. When multiple training images are available, as shown in Fig. 1, we can construct one set of weaker classifiers for each sample. Correspondingly, the label of the test image can be determined by integrating the outputs of all weaker classifiers. We will not discuss in this paper the case of multiple training samples because numerous algorithms have been developed for this.

### Conclusions

In this paper, we propose an efficient multi-resolution spectral feature image-based 2DLDA ensemble algorithm for the one-sample-image-per-person problem of face recognition. Experimental results have demonstrated that our proposed method has a higher recognition accuracy and robustness than some recently reported methods. Further, the experimental results also indicate that, for the proposed method, the larger the image size, the higher the recognition rates will be, and vice versa. In addition, pre-filtering is found to be an important step in the MR_2DLDA method. Compared to the 2DLDA method, the computation time required by the proposed method is higher. How to determine an efficient criterion to select a subset of the filters so as to reduce the computation burden while maintaining the performance level, is to be investigated in our future work.
